# The Choice of Anticoagulant Influences the Characteristics of Bone Marrow Aspirate Concentrate and Mesenchymal Stem Cell Bioactivity In Vitro

**DOI:** 10.1155/2022/8259888

**Published:** 2022-07-22

**Authors:** Ryan C. Dregalla, Jessica Ann Herrera, Lucanus Steven Koldewyn, Edward Jeffery Donner

**Affiliations:** ^1^Elite Regenerative Stem Cell Specialists, LLC, 4795 Larimer Parkway, Johnstown, CO 80534, USA; ^2^R&D Regenerative Laboratory Resources, LLC, 4795 Larimer Parkway, Johnstown, CO 80534, USA; ^3^Colorado Spine Institute, PLLC, 4795 Larimer Parkway, Johnstown, CO 80534, USA

## Abstract

Bone marrow aspirate concentrate (BMC) is commonly used as a therapeutic agent to resolve orthopedic injuries, using its unique cellularity to reduce inflammation and prime the region for repair. The aspiration of the bone marrow is performed using either sodium citrate (SC) or heparin sodium (HS) as an anticoagulant and processed via centrifugation to concentrate the cellular constituents. To date, the consideration of the impact of the two commonly used anticoagulants on the mesenchymal stem/stromal cell (MSC) population has been overlooked. The current study assesses the differences in the BMCs produced using 15% SC and HS at 1,000 U/mL or 100 U/mL final *v*./*v*. as an anticoagulant using in vitro metrics including total nucleated cell counts (TNC) and viability, the ability for mesenchymal stromal/stem cells (MSCs) to establish colony-forming units with fibroblast morphology (CFU-f), and cytokine expression profile of the MSC cultures. Our findings demonstrate that HS-derived BMC cultures result in higher CFU-f formation and CFU-f frequency at both concentrations assessed compared to SC-derived BMC cultures. In addition, there were significant differences in 27% (7 of 26) of the cytokines quantified in HS-derived BMC cultures compared to SC-derived BMC cultures with implications for MSC plasticity and self-renewal.

## 1. Introduction

The use of bone marrow aspirate (BMA) and BMA concentrate (BMC) has become increasingly popular in treating or managing pain associated with orthopedic and musculoskeletal conditions [1–3] and as an adjunct in surgical procedures [4, 5] to modulate the microenvironment of chronically inflamed/diseased tissues [1]. The benefit of BMC is thought to be associated with mesenchymal stem/stromal cell (MSC) populations, which are unique in their hematopoietic regulatory role(s) [6–8]. Clinically, the overall quality of BMC is typically associated with three metrics: (1) the total nucleated cell count (TNC), (2) viability, and (3) colony-forming units with a fibroblast morphology (CFU-f); CFU-f counts serve as a proxy for MSC counts. The rationale for TNC quantitation is that MSCs occur at a low frequency amongst the nucleated cells (~0.01% in newborns, ~0.0004% by 30 years of age, and ≤0.00025% at age 50 and older [9]) and therefore, higher TNC counts purportedly would result in more CFU-fs. However, this logic has recently been contested [10, 11]. While the role of other cell types unique to the bone marrow including CD34^+^ hematopoietic progenitor/precursor [12] cells and platelets [13] has been proposed as contributors to the therapeutic value of BMC, the value of MSCs in the BMC remains a pinnacle.

Techniques in the harvesting, processing, and dosage/volume of BMC are recognized as being inconsistent and problematic when determining the value of the orthobiologic. The harvesting of bone marrow aspirate can be commonly achieved using either a sodium citrate (SC) formulation (including acid citrate and dextrose containing formulations) or heparin sodium (HS). However, the influence of the anticoagulant selected has received little attention and manufacturers of BMA processing devices to create BMC suggest that the use of either anticoagulant is acceptable. Mechanistically, SC and HS function differently as anticoagulants. Citrate, the active anticoagulant in SC, is a divalent cation chelating compound, acting as an anticoagulant principally by reducing the availability of divalent ions in the extracellular environment (i.e., Ca^2+^ and Mg^2+^), which are major players in platelet activation, thrombin activity, and the formation of a thrombus/fibrin clot [14]. In contrast, heparin is a naturally synthesized highly sulfated glycosaminoglycan (GAG) which functions as an anticoagulant primarily via complexing with antithrombin III, resulting in the inhibition of thrombin enzymatic activity [15]. However, HS is a diverse GAG with several biological roles beyond functioning as an anticoagulant [16], including anti-inflammatory effects [17], which may influence cellular function downstream of BMA collection.

The aim of the present in vitro study was to determine whether SC and HS differentially influence BMC quality, using TNC, viability, and CFU-f as metrics, along with cytokine quantification in BMC in vitro cultures to assess potential differences in cell expression profiles resulting from the BMC processing in each anticoagulant. Donor-matched BMAs were collected using clinically relevant doses of SC (15% final *v*./*v*.) [18] and HS (1,000 U/mL and 100 U/mL final *v*./*v*.) [19–21]. Here, we find that the brief exposure of the BMAs to each respective anticoagulant during the processing to BMC results in distinct differences in the quality of BMC products.

## 2. Materials and Methods

### 2.1. Donor Sample Acquisition, Consent, and Demographics

All donors were patients scheduled for BMC intervention for a musculoskeletal condition requiring at least two BMA draws (at 50 mL per draw). Those who participated in the present study consented to donate specimens ≤ 0.5 mL of final BMC product derived from the respective anticoagulants (8.0-10 mL total per BMC, described below) for in vitro analysis. The patient treatment was in no way altered as a result of consenting to donate BMC specimens. Series 1 contained 8 females and 6 males with ages ranging from 27 to 76 years with an average age of 54. Series 2 contained 8 females and 5 males with ages ranging from 34 to 77 years with an average age of 61.

### 2.2. Harvest Preparation and Procedure

For all donors participating in the study, two 50 mL BMA draws were performed where each draw contained one of the two respective anticoagulants. Syringes were labeled arbitrarily as to blind the physician from the identity of the anticoagulant. For each donor, the bone marrow was aspirated bilaterally from the posterior iliac spine; anticoagulant used for the first aspiration was randomized. SC (sodium citrate 40 mg/mL stock solution, compounded Fagron Sterile Services, USA) was used in all cases at 15% final *v*./*v*. of the stock solution and HS (heparin sodium, Fresenius Kabi, USA) was used at two separate concentrations, 1,000 units/mL (U/mL) and 100 U/mL final *v*./*v*. (Series 1 and 2, respectively); 1 mg HS is ~120-140 U/mL [22]. All syringes and 11-gauge trocars were briefly rinsed/coated with HS solution before anticoagulants were loaded prior to BMA aspiration.

#### 2.2.1. Series 1

For all donors (*n* = 14), a total of 7.5 mL SC solution was added to a 60 mL syringe. In the 1,000 U/mL HS series, 5 mL of 10,000 U/mL was added to a 60 mL syringe. An additional 2.5 mL of sterile saline was added to the HS-containing syringe to adjust the final volume to 7.5 mL, matching the volume of the SC-containing syringe. During aspiration, syringes were filled to 50 mL with BMA.

#### 2.2.2. Series 2

In all donors (*n* = 13), HS was used at 100 U/mL final *v*./*v*.; SC remained at 15% final *v*./*v*. Here, 1 mL of 5,000 U/mL HS was added to a 60 mL syringe. Bone marrow aspirations resulted in 42.5 mL BMA in the SC-containing syringe and 49 mL of BMA in the HS-containing syringe. Volumes were not equalized using saline as performed previously in Series 1 in order to assess the differences between the anticoagulants using typical clinical practices.

To produce BMC in each series, BMAs were loaded into sterile 50 mL conical tubes and centrifuged at 2,200 relative centrifugal force (RCF) for 8 minutes. Buffy coats were collected and recentrifuged at 2,200 RCF for 5 minutes. The buffy coat was collected, avoiding disruption of the red blood cell interface. The buffy coat collected was then volumed up to a total of 8-10 mL (final volume) with residual plasma containing the respective anticoagulant.

### 2.3. Total Nucleated Cell Quantification

For BMAs and BMCs, 10 *μ*L from each sample was diluted 1 : 100 in 0.1% Triton X-100 (to permeabilize nucleated cells) containing 3.61 *μ*M 4′,6-diamidino-2-phenylindole (DAPI). Immediately, 10 *μ*L of the stained specimen was loaded onto a Neubauer hemocytometer/counting chamber. Images of each of the four 1 mm × 1 mm counting chamber grids were captured in phase contrast and under fluorescence at 104x total magnification using an AMG EVO FL microscope and “DAPI” light cube (357/44BP nm excitation; 447/60BP nm emission). Phase contrast and fluorescent images were overlayed for each of the four grids, and nucleated (DAPI^+^) cells were scored. Nucleated cell counts were determined based on the average count across the four fields scored adjusted for the respective dilution factor and reported per milliliter of BMA/BMC.

### 2.4. Cell Viability

For BMAs and BMCs, 10 *μ*L from each sample was diluted 1 : 100 in phosphate-buffered saline (Gibco, Cat. # 10010-023) containing 4.02 *μ*M calcein, acetoxymethyl ester (calcein-AM), and 3.61 *μ*M DAPI at cell impermeant levels as previously described [23]. The cell suspension was incubated at 37°C for 30 minutes then briefly vortexed. 10 *μ*L of the stained specimen was loaded onto a Neubauer hemocytometer/counting chamber. Images were captured, and overlays produced as described above with the addition of a third channel to detect calcein fluorescence via the GFP light cube (470/22 nm excitation; 510/22 nm emission). Counts from the four 1 mm × 1 mm grids were averaged, and viability is reported as a percentage where viable cells = calcein^+^/DAPI^−^ and dead cells = calcein^+^/DAPI^+^ or calcein^−^/DAPI^+^.

### 2.5. Colony-Forming Unit with Fibroblast Morphology Assay

Each BMC was introduced to culture immediately after processing. A total of 100 *μ*L of each BMC was added to 5 mL cell culture medium (Dulbecco's Modified Eagles Medium (Gibco, Cat. # 10567-014)) supplemented with 10% fetal bovine serum (Peak Serum, Cat. # PS-FB2) and 0.6 U/mL HS (to prevent medium solidification) in a 25 cm^2^ cell culture flask to establish adherent cell culture. Cultures were maintained at 37°C, 5% CO_2_. At day 5, medium was aspirated and cultures were washed with phosphate-buffered saline (PBS) (Gibco, Cat. # 10010-023) to remove nonadherent cells and fresh media were added to cultures. Cultures were maintained for 12-14 days and CFU-fs were quantified. As MSC populations are heterogenous and may proliferate at different rates [24], tightly clustered cell groupings containing ≥25 cells with fibroblast morphology were scored as colonies. CFU-f frequency was determined by dividing the CFU-f count/milliliter by the TNC count/milliliter, resulting a percentage of CFU-f forming cells amongst the initial cell population.

### 2.6. Protein Quantification Assay

Following processing, SC and HS-BMC were plated consistent with the methods in Series 2 and cultured as described in the CFU-f assay. Immediately after plating and brief mixing, 100 *μ*L of media was sampled (day 0) and again at days 5 (medium sampled prior to PBS wash and medium replacement) and 12. Medium samples were centrifuged at 10,000 RPM in a microcentrifuge for >30 seconds to remove cells/debris, and the supernatant was collected and stored at -20°C until assayed for cytokines. Cytokines were assayed using microbead arrays. Each prevalidated array was purchased from BioLegend (Cat. # 740180, Cat. # 740502). Samples were processed and analyzed per manufacturer's instructions. Beads were assayed using a Beckman Coulter CytoFLEX S; analysis of all fcs files to determine protein concentrations was done via BioLegend Qognit cloud-based software.

### 2.7. Statistical Analysis

In Series 1 and 2, all SC and HS preparations were donor matched and comparisons for TNC counts/milliliter, viability, CFU-f counts/milliliter, and CFU-f frequency were done via a paired two-tailed *t*-test. Comparisons between HS-BMC preparations containing 100 U/mL and 1,000 U/mL were not donor matched, and statistical analysis was performed via an unpaired two-tailed *t*-test for the metrics described above. Comparison of cytokine concentrations differed between growth factors and immunomodulators. For growth factor comparisons between donor-matched SC and HS-BMC cultures, it was hypothesized that HS-growth factor complexes would extend the protein half-life in vitro (described in Discussion). Therefore, a one-tailed paired *t*-test was used at each time point. As the influence of HS may be anti- or proinflammatory, comparison between SC and HS culture concentrations for immunomodulating factors was done using a paired two-tailed *t*-test at each time point. Confidence intervals were set to >95% for all analyses. Statistical analysis for all tests was performed via GraphPad Prism Software v9.3.1.

## 3. Results

### 3.1. Series 1: 15% SC Compared to 1,000 U HS/mL

#### 3.1.1. SC and HS Result in Differing TNC Counts in Final BMC with Equal Starting Volumes of BMA

**Figure 1:**
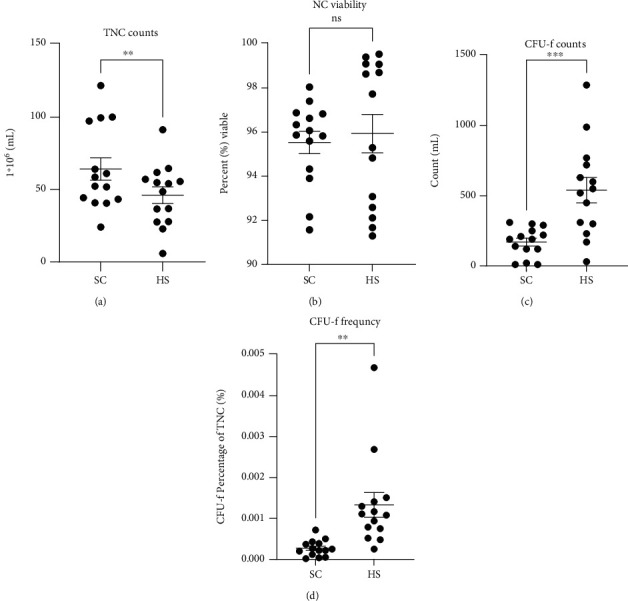
Comparison of BMC characteristics derived from 15% sodium citrate (SC) and 1,000 U/mL heparin sodium (HS) (Series 1): (a) total nucleated cell (TNC) count per milliliter, (b) nucleated cell (NC) viability, (c) colony-forming units with fibroblast morphology (CFU-f) counts per milliliter, and (d) frequency of CFU-f amongst the TNC population. Graphs display the population mean and standard error of the mean. ns: not significant, ^∗^*P* < 0.05, ^∗∗^*P* < 0.01, and ^∗∗∗^*P* < 0.001.

In comparisons between SC and 1,000 U/mL HS, TNC counts per milliliter were significantly higher in SC-BMCs (*P* = 0.0025, Figure 1(a)). Here, the average TNC was 64.20∗10^6^/mL (+/-7.72) and 46.02∗10^6^/mL (+/-5.75) for SC- and HS-BMC TNCs, respectively.

#### 3.1.2. Cell Viability Is Not Significantly Impacted by SC or HS Postprocessing

Immediately after processing, viability was not significantly impacted by the anticoagulant selected (*P* = 0.5886, Figure 1(b)). Viability was on average 95.53% (+/-0.51) and 95.93% (+/-0.87) for SC- and HS-BMCs, respectively.

#### 3.1.3. CFU-f Count and Frequency Differ between SC and HS-BMC Products

CFU-fs were scored for paired SC- and HS-BMC products at matched time points. Per milliliter, HS-BMCs showed a significant increase in CFU-fs compared to SC-BMCs, averaging 540 CFU-f/mL (+/-90) and 170 CFU-f/mL (+/-27.91), respectively (*P* = 0.0006, Figure 1(c)). To account for the difference in TNCs plated, the CFU-f frequency relative to the respective TNC was assessed. Mirroring the CFU-f count results, the CFU-f frequency was significantly elevated in HS-BMCs (0.0013% (+/-0.0003)) compared to SC-BMCs (0.00027% (+/-0.00005) (*P* = 0.0027, Figure 1(d)).

### 3.2. Series 2: 15% SC Compared to 100 U/mL HS

#### 3.2.1. SC and HS BMA Collections without Dilution Result in Similar TNC Counts in Final BMC

**Figure 2:**
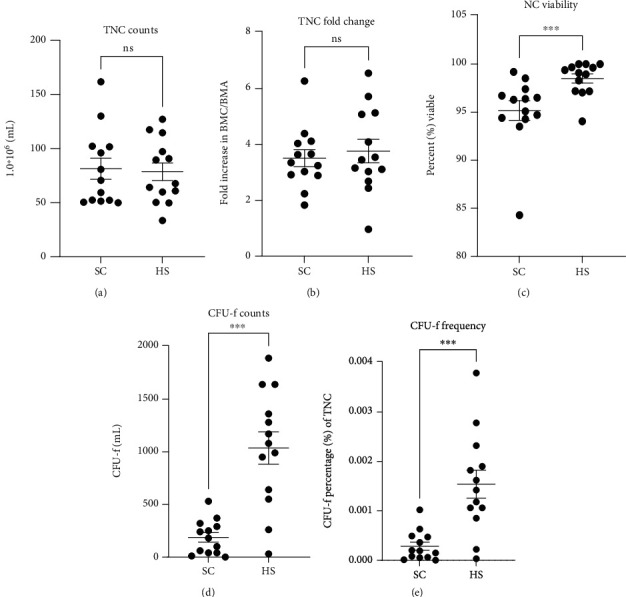
Comparison of BMC characteristics derived from 15% sodium citrate (SC) and 100 U/mL heparin sodium (HS) (Series 2): (a) total nucleated cell (TNC) count per milliliter, (b) fold concentration of TNC count/milliliter in BMC over initial BMA, (c) nucleated cell (NC) viability, (d) colony-forming units with fibroblast morphology (CFU-f) counts per milliliter, and (e) frequency of CFU-f amongst the TNC population. Graphs display the population mean and standard error of the mean. ns: not significant, ^∗^*P* < 0.05, ^∗∗^*P* < 0.01, and ^∗∗∗^*P* < 0.001.

To simulate clinical practice where saline dilution is not a factor and to investigate the feasibility of lower concentration of HS (final U/mL), 42.5 mL of BMA was collected in 7.5 mL SC (50 mL total) and 49.0 mL BMA into 1 mL HS solution (described in Materials and Methods). Clotting was not observed in either case. In the present series, TNC was documented for BMA and BMC in SC and HS conditions allowing for fold change in the final BMC to be calculated. In contrast to the TNC data collected in Series 1, SC-BMC and 100 U/mL HS-BMC resulted in similar TNC count/milliliter resulting in 81.90∗10^6^ (+/-9.86) and 79.10∗10^6^ (+/-8.22), respectively (*P* = 0.8214, Figure 2(a)). Additionally, calculating the fold change in BMC TNC over the starting BMA revealed no difference in the SC and HS groups (3.51 (+/-0.31) and 3.76 (+/-0.42) fold increase, respectively) (Figure 2(b)).

#### 3.2.2. Viability in SC-BMC Differs from HS-BMC

Following processing, viability in SC-BMC was significantly reduced compared to HS (100 U/mL) BMCs. In SC-BMC, viability was 95.18% (+/-1.02) whereas HS-BMC viability was 98.50% (+/-0.48) (*P* = 0.0008, Figure 2(c)).

#### 3.2.3. CFU-f Count and Frequency Differ between SC and HS-BMC Products

**Figure 3:**
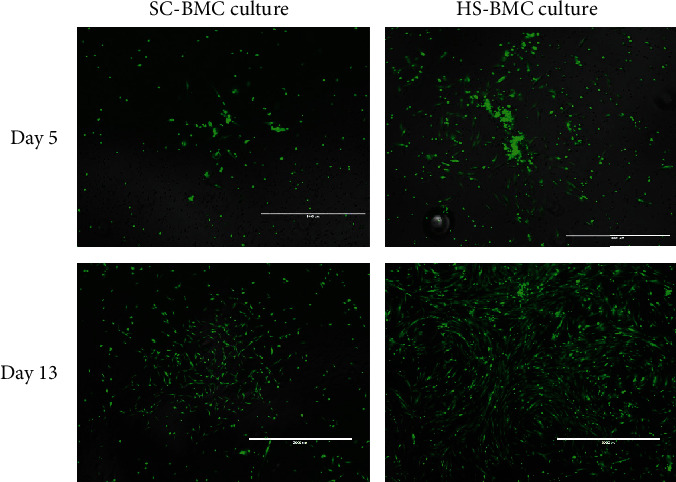
Calcein-AM-stained CFU-fs in culture from sodium citrate- (SC-) and heparin sodium- (HS-) derived BMCs at day 5 and day 13. BMCs imaged are donor matched. Day 5 micrographs were captured at 104x total magnification, scale bar = 1,000 *μ*m. Day 13 micrographs were captured at 52x total magnification, scale bar = 2,000 *μ*m.

Assessment of CFU-f/milliliter in donor-matched SC and 100 U/mL HS-BMCs mirrored the results observed in Series 1. Here, SC-BMC CFU-f counts were 187 per milliliter (+/-45.25) on average which was significantly lower compared to the 1037 (+/-153) CFU-fs per milliliter in the HS-BMCs (*P* = 0.0001, Figure 2(d)). Similarly, the frequency of CFU-f forming cells amongst the TNC was 0.0002854% (+/-0.000083) for SC-BMC cultures and 0.0015% (+/-0.00028) for HS-BMC cultures (*P* = 0.0007, Figure 2(e)). Overall, HS-BMC cultures produced more robust CFU-fs compared to HS-BMC cultures (Figure 3).

### 3.3. Comparisons between 1,000 U/mL and 100 U/mL HS

#### 3.3.1. BMCs Containing HS at 100 U/mL and 1,000 U/mL Differ in TNC Counts

**Figure 4:**
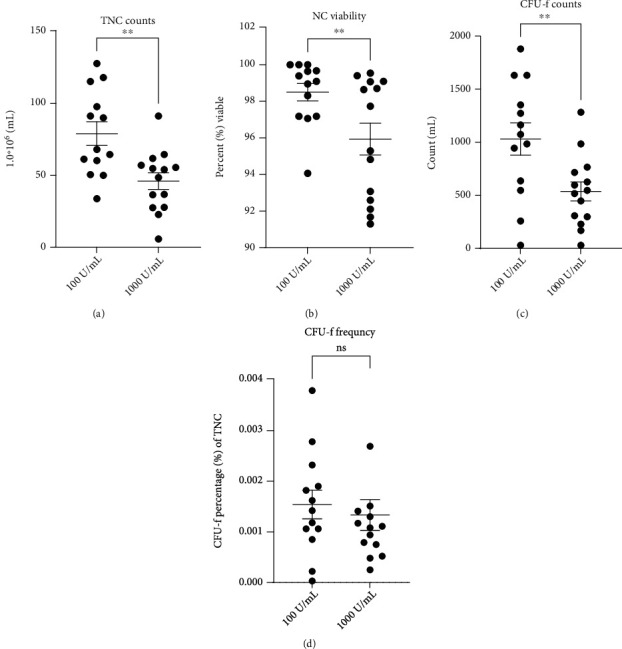
Comparison of BMC characteristics derived from 100 U/mL heparin sodium (100 U/mL) and 1,000 U/mL heparin sodium (1,000 U/mL). Comparisons between the respective BMCs: (a) total nucleated cell (TNC) count per mL, (b) nucleated cell (NC) viability, (c) colony-forming units with fibroblast morphology (CFU-f) counts per mL, and (d) frequency of CFU-f amongst the TNC population. Graphs display the population mean and standard error of the mean. ns: not significant, ^∗^*P* < 0.05 and ^∗∗^*P* < 0.01.

The TNC counts in BMCs containing 1,000 U/mL HS were significantly lower compared to those containing 100 U/mL. On average, the TNC counts were 46.02∗10^6^ (+/-5.75) and 79.10∗10^6^ (+/-8.22), respectively (*P* = 0.0013, Figure 4(a)).

#### 3.3.2. Higher HS Concentrations Impact Cell Viability in BMCs

In the 1,000 U/mL HS-BMCs, the average viability was 95.93% (+/-0.87) compared to 98.50% (+/-0.48) in the BMCs containing 100 U/mL HS (Figure 4(b)). The difference was significant between the two groups (*P* = 0.0091).

#### 3.3.3. CFU-f Counts Are Increased in BMCs with Lower HS Concentrations

CFU-f counts in BMCs containing 100 U/mL HS averaged 1034 CFU-fs/mL (+/-153) of BMC, which was significantly higher compared to BMCs containing 1,000 U/mL which averaged 540 (+/-90) CFU-fs/mL of BMC (*P* = 0.0043, Figure 4(c)). However, the difference in the frequency of CFU-fs with respect to the TNC counts was not significant between the two HS groups (*P* = 0.3113, Figure 4(d)).

### 3.4. Cytokine/Chemokine Profiles Differ between SC and HS-BMC Cultures

The differences in HS and SC cultures, particularly with respect to CFU-f formation of the 12-day culture period, indicated that there may be an increase in the concentration of soluble factors in HS cultures compared to SC cultures. As the SC and 100 U/mL HS groups were similar in initial TNC counts, donor-matched draws were assayed as described in Series 2. Cytokine/chemokines were assayed at days 0, 5, and 12 to detect dynamic changes.

Of the growth factors assayed, there was no significant difference at any time point between SC and 100 U/mL HS groups for G-CSF, EGF, EPO, FGFb/FGF-2, GM-CSF, M-CSF, PDGF-BB, SCF, or TGF-*α* (data not shown). Of the inflammatory mediator assayed, there was no significant difference at any time point between the groups for IL-12p70, TNF-*α*, IL-4, IL-10, IL-1*β*, arginase, CCL17/TARC, IRAP, IFN-*γ*, or CXCL10/IP-10 (data not shown). Cytokines where differences between SC and HS cultures were significant within the time course are below.

### 3.5. Growth Factors

#### 3.5.1. Angiopoietin-2

**Figure 5:**
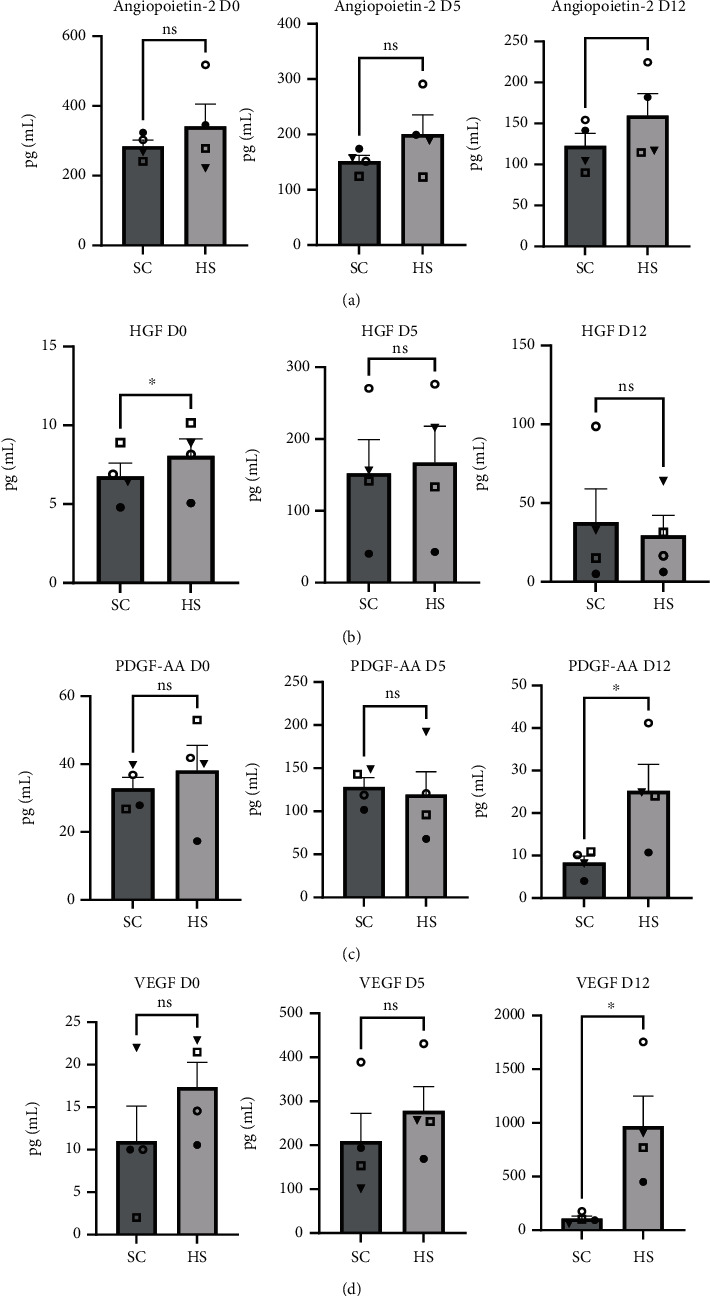
Growth factor concentrations in cell culture media with BMCs containing 15% sodium citrate (SC) or 100 U/mL heparin sodium (HS) at day 0 (D0), day 5 (D5), and day 12 (D12): (a) angiopoeitin-2, (b) hepatocyte growth factor (HGF), (c) platelet-derived growth factor dimer AA (PDGF-AA), and (d) vascular endothelial growth factor (VEGF). Graphs display the population mean and standard error of the mean; shapes indicate donor-matched BMC products. ns: not significant, ^∗^*P* < 0.05.

Levels of angiopoietin-2 did not differ between the groups at day 0 or day 5. By day 12, the SC group and HS group averaged 122.6 pg/mL (+/-15.1) and 159.6 pg/mL (+/-26.75), respectively, where HS-BMC cultures showed a significant increase over SC-BMC cultures (*P* = 0.0303, Figure 5(a)).

#### 3.5.2. HGF

Following BMC processing and plating (day 0), HGF concentrations were significantly elevated in HS-BMC cultures at 8.06 pg/mL (+/-1.08) compared to SC cultures 6.76 pg/mL (+/-0.084) (*P* = 0.0301, Figure 5(b)). Latter time points showed increased levels of HGF compared to day 0 in both culture groups though did not differ from one another.

#### 3.5.3. PDGF-AA

At day 0 and day 5, there was no difference in levels of PDGF-AA between the SC- and HS-BMC cultures. By day 12, levels of PDGF-AA in HS-BMC cultures were 25.22 pg/mL (+/-6.24), which was significantly elevated compared to SC-BMC cultures averaging 8.34 pg/mL (+/-1.55) (*P* = 0.023, Figure 5(c)).

#### 3.5.4. VEGF

Concentrations of VEGF were not significantly different between SC- and HS-BMC cultures at day 0 and day 5. At day 12, HS-BMC culture levels of VEGF were significantly elevated over SC-BMC cultures, where levels were 970.60 pg/mL (+/-278) and 109.20 pg/mL (+/-23.97), respectively (*P* = 0.022, Figure 5(d)).

### 3.6. Immunomodulators

#### 3.6.1. IL-6

**Figure 6:**
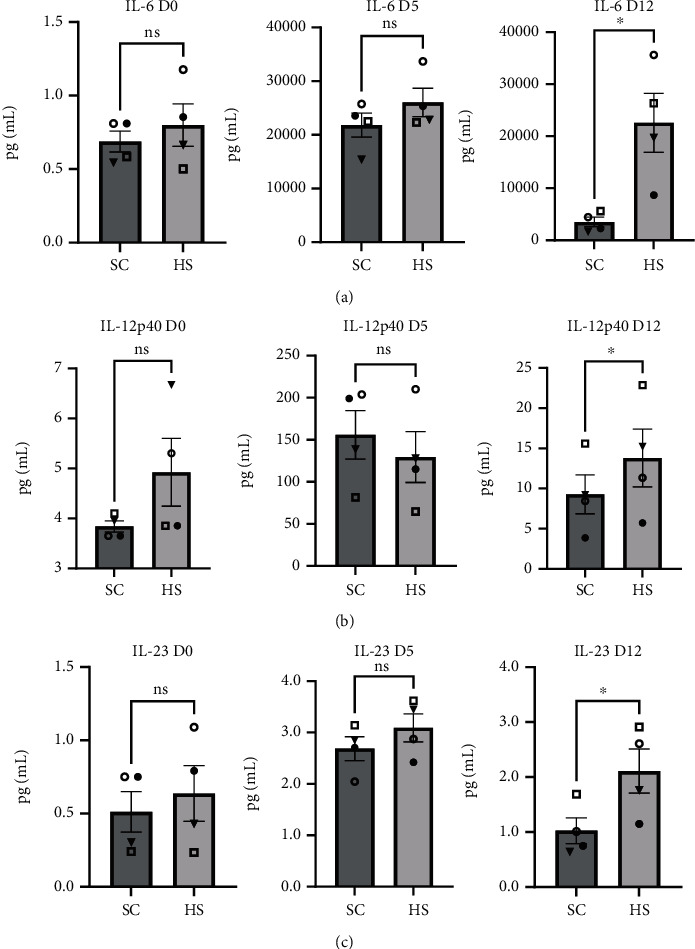
Immunomodulating cytokine concentrations in cell culture media with BMCs containing either sodium citrate (SC) or heparin sodium (HS) at day 0 (D0), day 5 (D5), and day 12 (D12): (a) IL-6, (b) IL-12p40, and (c) IL-23. Graphs display the population mean and standard error of the mean; shapes indicate donor-matched BMC products. ns: not significant, ^∗^*P* < 0.05.

There was no difference between the SC- and HS-BMC cultures in IL-6 concentrations at the first two time points. At day 12, IL-6 levels were significantly elevated in HS-BMC cultures (22,597 pg/mL +/- 5,666) compared to SC-BMC cultures (3,522 pg/mL +/- 915.8) (*P* = 0.0331, Figure 6(a)).

#### 3.6.2. IL-12p40

At day 0 and day 5, there was no difference in the levels of IL-12p40 between SC- and HS-BMC cultures. At day 12, IL-12p40 levels were 13.80 pg/mL (+/- 3.61) in HS-BMC cultures, which was significantly elevated compared to SC-BMC cultures averaging 9.28 pg/mL (+/- 2.41) (*P* = 0.039, Figure 6(b)).

#### 3.6.3. IL-23

No difference in IL-23 levels was observed between SC and HS-BMC cultures at day 0 or day 5. HS-BMC cultures at day 12 showed significantly higher levels of IL-23 (2.11 pg/mL +/- 0.40) compared to SC-BMC cultures (1.02 pg/mL +/- 0.23) (*P* = 0.023, Figure 6(c)).

## 4. Discussion

The objective of the present study was to assess innate differences in BMC preparations containing SC or HS, both of which are used for bone marrow aspiration [21, 25]. Our study evaluated two separate concentrations of HS, where BMA was collected into HS solutions resulting in clinically relevant final concentrations of HS at 1,000 U/mL and 100 U/mL of BMA [20, 21], representing Series 1 and Series 2, respectively.

In Series 1, SC aspirates resulted in higher TNC counts compared to HS with no difference in cell viability. This data suggests that the use of SC consistently results in a higher TNC count compared to volume-matched HS solutions in the final BMC product. However, at this phase of the study, initial BMA TNCs were not quantified; therefore, it is unknown if this result is due to a higher initial TNC in the BMA or if the buffy coat was of higher quality in the SC-BMC compared to the HS-BMC. As both BMC products were plated based on volume, not TNC, we anticipated to observe elevated CFU-f counts in the SC-BMC, without a difference in the frequency of CFU-fs between the SC- and HS-BMC groups. In contrast to the anticipated result, HS-BMCs were significantly elevated in both CFU-f counts per milliliter and frequency amongst the TNCs compared to SC-BMC cultures. This striking difference is a strong indicator that HS is providing an innate biochemical benefit to the MSC population.

To simulate clinical practice, where the clinician would not dilute HS solutions to match the SC volume at 15% final *v*./*v*. and to examine lower doses of HS, Series 2 in the study was designed. Regardless of the larger starting BMA volume in the HS group compared the SC group in this series (49.0 mL*v*.and 42.5 mL BMA, respectively), there was no difference in the TNC count in BMAs and BMCs derived from the two anticoagulants. Furthermore, the fold increase in BMC TNC count over the respective BMA baseline did not differ between the SC and HS groups. Taken together, the data demonstrates that SC and HS at the volumes used in Series 2 result in equivalent BMA and BMC products when using TNC counts as a metric.

Despite the equivalency in TNC counts per milliliter in the SC and HS groups in Series 2, the difference in CFU-f counts and frequency was significantly higher in the HS group. Consistent with the findings in Series 1, this suggests that HS is providing an (unknown) advantage to MSCs. It is important to consider that the volume plated immediately after processing is diluted 50-fold into the culture media (100 *μ*L BMC in 5.0 mL media), which would suggest that the influence of the anticoagulant is nullified in culture and therefore, the impact of the two respective anticoagulants is likely occurring during processing (discussed below).

Comparing 100 U/mL and 1000 U/mL HS conditions revealed several interesting differences in BMC preparations. Principally, this was with respect to CFU-f counts and frequency amongst the TNC counts. It is practical to presume that TNC and CFU-f counts would fluctuate between the two HS groups, as the BMA draw volumes differed, which was the case. However, the CFU-f frequency did not differ between 100 U/mL and 1,000 U/mL HS-BMCs, demonstrating that the quality of the BMA/BMC from the two respective groups was equivalent.

To examine if the secretome of BMC cells in culture differs between the SC- and HS-BMC preparations, 26 cytokines/chemokines were assayed in the cell culture media. Of the cytokines assessed, those which showed significant increases in HS-BMC cultures over SC-BMC cultures were angiopoietin-2, HGF, PDGF-AA, VEGF, IL-6, IL-12p40, and IL-23. This is evidence of differences between SC- and HS-BMC culture secretomes though it is unclear as to how important (or not) these few factors are in contributing to the large differential in CFU-f count and frequency between the anticoagulant conditions. Each cytokine is known to interact with heparin [26–29], potentially extending their half-life by protecting from proteolytic degradation or strengthening their signaling capability via promoting extended interactions with the cell surface receptor [30–33]. Interestingly, some of the cytokines assayed known to interact with HS and promote MSC proliferation (e.g., bFGF/FGF-2 [34, 35]) did not show significant differences between SC- and HS-BMC cultures at any time point. While no difference was observed in the concentration of several factors assayed in the respective cultures, we cannot rule out the advantage of heparin interacting with these growth factors and promoting ligand-receptor interactions, extending intracellular signal transduction, and increasing proliferation and CFU-f formation.

Of the cytokines that differed in concentrations between the conditions, VEGF and IL-6 levels continued to increase at each subsequent time point in HS-BMC cultures, suggesting active expression in vitro. This is intriguing, as VEGF expression via mammalian MSCs has been proposed to be directly related to MSC potency and proliferation [36, 37]. It has been suggested that MSCs do not secrete adequate VEGF level in vitro for autocrine signaling and that MSCs transduced to express VEGF demonstrate increased proliferation, making our findings even more intriguing provided the magnitude of difference between SC and HS cultures, which has significant clinical implications. While IL-6 is commonly viewed as a proinflammatory protein, the fact that IL-6 has both anti- and proinflammatory modulating activity is often overlooked [38]. IL-6 is a key player in retaining MSC function as a progenitor cell and is ancillary in MSC proliferation. An in vitro study by Pricola et al. demonstrated that using 10 ng/mL of IL-6 during in vitro culture of human MSCs increased cellular proliferation and retained MSC “stemness”/plasticity. In addition, the study showed that siRNA-mediated knockdown of IL-6 expression via MSCs in vitro significantly reduced proliferation [39]. These results strongly support our observation with respect to IL-6 concentrations and CFU-f formation in HS-containing BMC cultures. We speculate that HS influences an array of cytokine-receptor interactions which alters intracellular signaling cascades and cell expression profiles which includes VEGF and IL-6.

While we found ample evidence that HS at the very least appears to stabilize numerous cytokines under these experimental conditions and increases the expression of others, it is important to recognize where and when this is occurring. As the BMC products are each heavily diluted in culture medium (containing low levels of HS in all cases) when plated, the actual difference between the two preparations is unlikely due to culture conditions but rather the influence of HS during processing. Typically, processing requires 30-40 minutes and between BMA collection and final injection; the time elapsed may be 60-90 minutes. During this time, exogenous HS used as an anticoagulant is actively complexing with cytokines and cell surface receptors. Once the final BMC product is produced, these interactions are plausibly stable and carry over into culture.

We postulate that the mechanisms by which the HS results in an increase in CFU-f counts and CFU-f frequency compared to SC in vitro are via the following: (1) HS-cell receptor complexes exacerbate cytokine interactions with the receptor, resulting in extended intracellular signal transduction and resulting in proliferation and altered expression profiles (VEGF/IL-6 production), and (2) cytokine interaction with HS results in extended half-lives. The ability of HS to promote and extend cytokine-receptor interactions may be an important influence on cytokines where no significant difference in concentrations was reported though it may have been imperative in the dramatic difference in the CFU-f data between SC and HS BMC cultures. Translationally, this would indicate that HS-processed BMC is favorable to human MSCs in their ability to proliferate and populate a location outside of the bone marrow and alter the foreign microenvironment, feasibility leading to improved and more consistent clinical outcomes. However, we cannot rule out the possibility that the anticoagulants used impact MSC concentration and/or survival in the final BMC product, resulting in the difference in CFU-f metrics reported here. It is possible that MSC-specific surface marker assessment of the BMCs via flow cytometry prior to plating would have at least partially addressed this question though, no universally accepted panel exists to identify the various subpopulations of MSCs in the marrow [21, 40–43].

## 5. Conclusion

Our results clearly illustrate that HS and SC can be used as anticoagulants in BMA collection to acquire similar TNC counts/milliliter and cell viability in BMC products. However, heparinized-BMC products showed an increase in CFU-f counts/milliliter and CFU-f frequency compared to SC-BMC preparations. Further, HS appears to impact bone marrow niche cytokine stability and possibly function in vitro, providing a potential mechanistic explanation for the differences in the CFU-f characteristics between SC- and HS-BMC cultures. These results suggest that the clinical use of BMC should incorporate HS as an anticoagulant as opposed to SC. However, future clinical studies to assess outcomes in SC- and HS-BMC preparation will be ancillary to further this postulation.

## Data Availability

The datasets used and/or analyzed during the current study are available from the corresponding author on reasonable request.

## Additional Points

*Clinical Significance*. The use of HS as an anticoagulant is favorable in BMC collection where MSC proliferation and the associated secretome are of therapeutic value to the clinician.

## References

[1] Chahla J., Dean C. S., Moatshe G., Pascual-Garrido C., Serra Cruz R., LaPrade R. (2016). Concentrated bone marrow aspirate for the treatment of chondral injuries and osteoarthritis of the knee: a systematic review of outcomes. *Orthopaedic Journal of Sports Medicine*.

[2] Gianakos A. L., Sun L., Patel J. N., Adams D. M., Liporace F. A. (2017). Clinical application of concentrated bone marrow aspirate in orthopaedics: a systematic review. *World Journal of Orthopedics*.

[3] Kim G. B., Seo M. S., Park W. T., Lee G. W. (2020). Bone marrow aspirate concentrate: its uses in osteoarthritis. *International Journal of Molecular Sciences*.

[4] Hernigou P., Poignard A., Beaujean F., Rouard H. (2005). Percutaneous autologous bone-marrow grafting for nonunions. Influence of the number and concentration of progenitor cells. *The Journal of Bone and Joint Surgery. American Volume*.

[5] Barber S. M., Radaideh M., Parrish R. (2018). Efficacy of autogenous bone marrow aspirate as a fusion-promoting adjunct to anterior cervical discectomy and fusion: a single center retrospective cohort study. *Cureus*.

[6] Crippa S., Bernardo M. E. (2018). Mesenchymal stromal cells: role in the BM niche and in the support of hematopoietic stem cell transplantation. *Hema*.

[7] Zorina T. D. (2021). New insights on the role of the mesenchymal-hematopoietic stem cell axis in autologous and allogeneic hematopoiesis. *Stem Cells and Development*.

[8] Zhang L., Mack R., Breslin P., Zhang J. (2020). Molecular and cellular mechanisms of aging in hematopoietic stem cells and their niches. *Journal of Hematology & Oncology*.

[9] Caplan A. I. (2007). Adult mesenchymal stem cells for tissue engineering versus regenerative medicine. *Journal of Cellular Physiology*.

[10] Cercone M., Greenfield M. R., Fortier L. A. (2021). Bone marrow concentrate mesenchymal stromal cells do not correlate with nucleated cell count or colony forming units. *Journal of Cartilage & Joint Preservation*.

[11] Muench L. N., Berthold D. P., Kia C. (2021). Nucleated cell count has negligible predictive value for the number of colony- forming units for connective tissue progenitor cells (stem cells) in bone marrow aspirate harvested from the proximal humerus during arthroscopic rotator cuff repair. *Arthroscopy*.

[12] Kubsik-Gidlewska A., Klupiński K., Krochmalski M., Krochmalski J., Klimkiewicz P., Woldańska-Okońska M. (2018). CD34+ stem cell treatment for knee osteoarthritis: a treatment and rehabilitation algorithm. *Journal of Rehabilitation Medicine-Clinical Communications*.

[13] Dregalla R. C., Herrera J. A., Donner E. J. (2021). Soluble factors differ in platelets derived from separate niches: a pilot study comparing the secretome of peripheral blood and bone marrow platelets. *Cytotherapy*.

[14] Lee G., Arepally G. M. (2012). Anticoagulation techniques in apheresis: from heparin to citrate and beyond. *Journal of Clinical Apheresis*.

[15] Linhardt R. J., Dordick J., Deangelis P., Liu J. (2007). Enzymatic synthesis of glycosaminoglycan heparin. *Seminars in Thrombosis and Hemostasis*.

[16] Lima M., Rudd T., Yates E. (2017). New applications of heparin and other glycosaminoglycans. *Molecules*.

[17] Mousavi S., Moradi M., Khorshidahmad T., Motamedi M. (2015). Anti-inflammatory effects of heparin and its derivatives: a systematic review. *Advances in Pharmacological Sciences*.

[18] Anz A. W., Hackel J. G. (2020). Bone marrow aspirate concentrate is equivalent to PRP for the treatment of knee OA at 1 year: response. *Orthopaedic Journal of Sports Medicine*.

[19] Jeyaraman M., Bingi S. K., Muthu S. (2022). Impact of the process variables on the yield of mesenchymal stromal cells from bone marrow aspirate concentrate. *Bioengineering*.

[20] Roger Y., Burmeister L., Hamm A. (2020). Heparin anticoagulant for human bone marrow does not influence in vitro performance of human mesenchymal stromal cells. *Cell*.

[21] Schafer R., De Baun M. R., Fleck E. (2019). Quantitation of progenitor cell populations and growth factors after bone marrow aspirate concentration. *Journal of Translational Medicine*.

[22] Prus R. M. (1974). Heparins and heparin units. *JAMA*.

[23] Dregalla R. C., Herrera J. A., Donner E. J. (2021). Red blood cells and their releasates compromise bone marrow-derived human mesenchymal stem/stromal cell survival in vitro. *Stem Cell Research & Therapy*.

[24] Cordeiro-Spinetti E., de Mello W., Trindade L. S., Taub D. D., Taichman R. S., Balduino A. (2014). Human bone marrow mesenchymal progenitors: perspectives on an optimized in vitro manipulation. *Frontiers in Cell and Development Biology*.

[25] Oliver K., Awan T., Bayes M. (2017). Single- versus multiple-site harvesting techniques for bone marrow concentrate: evaluation of aspirate quality and pain. *Orthopaedic Journal of Sports Medicine*.

[26] Ori A., Wilkinson M. C., Fernig D. G. (2011). A systems biology approach for the investigation of the heparin/heparan sulfate interactome. *The Journal of Biological Chemistry*.

[27] Mummery R. S., Rider C. C. (2000). Characterization of the heparin-binding properties of IL-6. *Journal of Immunology*.

[28] Hasan M., Najjam S., Gordon M. Y., Gibbs R. V., Rider C. C. (1999). IL-12 is a heparin-binding cytokine. *Journal of Immunology*.

[29] Garnier P., Mummery R., Forster M. J., Mulloy B., Gibbs R. V., Rider C. C. (2018). The localisation of the heparin binding sites of human and murine interleukin-12 within the carboxyterminal domain of the P40 subunit. *Cytokine*.

[30] Coombe D. R. (2008). Biological implications of glycosaminoglycan interactions with haemopoietic cytokines. *Immunology and Cell Biology*.

[31] Hachim D., Whittaker T. E., Kim H., Stevens M. M. (2019). Glycosaminoglycan-based biomaterials for growth factor and cytokine delivery: making the right choices. *Journal of Controlled Release*.

[32] Chiodelli P., Bugatti A., Urbinati C., Rusnati M. (2015). Heparin/heparan sulfate proteoglycans glycomic interactome in angiogenesis: biological implications and therapeutical use. *Molecules*.

[33] Sarrazin S., Lamanna W. C., Esko J. D. (2011). Heparan sulfate proteoglycans. *Cold Spring Harbor Perspectives in Biology*.

[34] Koledova Z., Sumbal J., Rabata A. (2019). Fibroblast growth factor 2 protein stability provides decreased dependence on heparin for induction of FGFR signaling and alters ERK signaling dynamics. *Frontiers in Cell and Development Biology*.

[35] Lee J. S., Kim S. K., Jung B. J., Choi S. B., Choi E. Y., Kim C. S. (2018). Enhancing proliferation and optimizing the culture condition for human bone marrow stromal cells using hypoxia and fibroblast growth factor-2. *Stem Cell Research*.

[36] Bhat S., Viswanathan P., Chandanala S., Prasanna S. J., Seetharam R. N. (2021). Expansion and characterization of bone marrow derived human mesenchymal stromal cells in serum-free conditions. *Scientific Reports*.

[37] Kong X., Zheng F., Guo L. Y. (2010). VEGF promotes the proliferation of bone marrow derived mesenchymal stem cells through ERK1/2 signal pathway. *Zhongguo Shi Yan Xue Ye Xue Za Zhi*.

[38] Scheller J., Chalaris A., Schmidt-Arras D., Rose-John S. (2011). The pro- and anti-inflammatory properties of the cytokine interleukin-6. *Biochimica et Biophysica Acta*.

[39] Pricola K. L., Kuhn N. Z., Haleem-Smith H., Song Y., Tuan R. S. (2009). Interleukin-6 maintains bone marrow-derived mesenchymal stem cell stemness by an ERK1/2-dependent mechanism. *Journal of Cellular Biochemistry*.

[40] Kandarakov O., Belyavsky A., Semenova E. (2022). Bone marrow niches of hematopoietic stem and progenitor cells. *International Journal of Molecular Sciences*.

[41] Li H., Ghazanfari R., Zacharaki D., Lim H. C., Scheding S. (2016). Isolation and characterization of primary bone marrow mesenchymal stromal cells. *Annals of the New York Academy of Sciences*.

[42] Mabuchi Y., Okawara C., Méndez-Ferrer S., Akazawa C. (2021). Cellular heterogeneity of mesenchymal stem/stromal cells in the bone marrow. *Frontiers in Cell and Development Biology*.

[43] Roson-Burgo B., Sanchez-Guijo F., del Cañizo C., de Las Rivas J. (2016). Insights into the human mesenchymal stromal/stem cell identity through integrative transcriptomic profiling. *BMC Genomics*.

